# The Impact of Accountability-Oriented Control Aspects of Variance Investigation on Budgetary Slack and Moderating Effect of Moral Development

**DOI:** 10.3389/fpsyg.2020.583643

**Published:** 2020-12-10

**Authors:** Deqiang Deng, Lana Yan Jun Liu, Subin Wen

**Affiliations:** ^1^College of Economics and Management, Nanjing Forestry University, Nanjing, China; ^2^Business School, Newcastle University, Newcastle upon Tyne, United Kingdom; ^3^Institute of Intelligent Management Accounting and Internal Control, Nanjing Audit University, Nanjing, China

**Keywords:** accountability-oriented control, variance investigation, external investigation, self-reporting, budgetary slack, moral development

## Abstract

Variance investigation (VI) has been identified as an effective mechanism to reduce budgetary slack at the *ex ante* budgeting stage. This paper focuses on two further research questions: (1) the extent to which two different accountability-oriented control aspects (i.e., external investigation and self-reporting) of VI affect budgetary slack and (2) the extent of the moderating effect of moral development on the relationship between these two accountability-oriented control aspects and budgetary slack. Our experimental results show that both external investigation and self-reporting can reduce the propensity of creating slack at the *ex ante* budgeting stage. More specifically, the effect of external investigation on reducing the propensity of creating budgetary slack is greater than that of self-reporting. This study further reveals that moral development moderates the effect of external investigation on budgetary slack. When comparing subordinates with low moral development with those with high moral development, our results show that the effect of external investigation on budgetary slack is stronger among the former group than the latter group. This study does not find any moderating effect of moral development on the relationship between self-reporting and budgetary slack. Our study sheds some new light on varying effects of two accountability-oriented control aspects of VI on budgetary slack, which are also moderated by different levels of subordinates’ moral development. These results may be considered in the design and implementation of management control systems.

## Introduction

Variance investigation (VI) is known as one of the management control mechanisms to reduce budgetary slack, due to its ability to relieve information asymmetry (Lambert, 1985) and to generate accountability pressure (Webb, 2002). VI is thus identified as one of the major budgeting designs and implementation mechanisms in the existing literature and practice (Sponem and Lambert, 2016). Majority of existing budgeting studies regard VI as a control mechanism of the *ex post* budgeting (implementation) stage, at which managers investigate variances between forecasts and actuals and assign responsibility for those variances to appropriate behavior subjects (Brownell, 1983; Hirst and Lowy, 1990; Emsley, 2001). However, VI can also generate a curbing effect if it is implemented during the *ex ante* budgeting (setting) stage and thus regarded as a specific control feature (Webb, 2002) due to its inherent accountability notion. According to Webb (2002, p. 362), “investigating large budget variances will generate accountability pressure to create less budget slack.” Furthermore, under his proposed theoretical framework, VI can generate accountability pressure onto subordinates participating at the *ex ante* budgeting stage and subsequently may effectively reduce subordinates’ propensity to create budget slack. It is therefore reasonable to regard Webb’s (2002) VI as part of accountability-oriented control systems (Merchant and Otley, 2006; Bergsteiner, 2012).

Accountability is a process in which individuals should be answerable to audiences, oneself included, for events that are associated with one’s identity and are relevant to salient prescriptions about how things should be (Schlenker and Weigold, 1989). It is generally understood that accountability is a complex and non-unitary construct with multiple phases and different aspects (Schlenker and Weigold, 1989; Schlenker, 1997; Lerner and Tetlock, 1999; Erdogan et al., 2004; DeZoort et al., 2006). Schlenker and Weigold (1989) describe how accountability evolves through four phases, including inquiry, accounting, judgment, and sanction^1^. The first two phases can be described as *ex ante* of an event, whereas the last two phases are linked with *ex post* of the event. Thus, it may be reasonable to align two specific control aspects of VI (external investigation and self-reporting^2^) as stipulated by Webb (2002) with the first two accountability phases, inquiry and accounting, respectively.

Further, Webb (2002) suggests that both external investigation and self-reporting can generate accountability pressure, which subsequently reduces subordinates’ propensity to create budgetary slack^3^ at the *ex ante* budgeting stage. It will be of interest to ascertain in detail these two specific control aspects of VI from a multiple phase perspective of an accountability process. We thus propose that (1) when significant budget variances are to be investigated by superior, namely, external investigation occurs, the level of budgetary slack of subordinates will decrease; (2) when the subordinates are to be required to indicate the causes of significant budget variances, namely, self-reporting occurs, the level of budgetary slack of subordinates will decrease.

It is worth noting that there are some differences between accountability pressure generated by external investigation and that by self-reporting, which may result in different impacts on budgetary slack. Subordinates’ perceived accountability pressure generated by external investigation comes from the supervisor’s investigation according to the VI policies and management rules (e.g., when there is a “significant” number of budgetary variances between subordinate’s actual capacity known by the supervisor and his/her submitted budgetary target). Therefore, the level of subordinates’ perceived accountability pressure generated by external investigation stems mainly from these external rules and the extent to which the external supervisor implements these rules. On the other hand, subordinates’ self-reporting is indicated in the external rules and only required when significant budgetary variances occur. The quantity and quality (such as authenticity and timeliness, etc.) about the cause of “significant” budgetary variances reported by subordinates are mainly affected by the subordinate’s self-identification and intrinsic principles. The latter is related with the process, means, or result of showing oneself to be a particular type of person (Schlenker and Weigold, 1989; Webb, 2002). It can be argued that subordinate’s perception to the level of accountability pressure generated by self-reporting stems mainly from intrinsic principles besides external rules. This is in line with self-determination theory, whereby external investigation can be regarded as a form of extrinsic motivation, in which behavior is initiated and regulated by contingencies external to the person. Self-reporting, on the other hand, can be identified as introjected regulation that motivates people to behave in line with the regulation within oneself and their fragile egos (Ryan, 1982; Gagné and Deci, 2005). Although external investigation and self-reporting are related with controlled motivation, self-reporting can stimulate more intrinsic motivation than external investigation. Intrinsic motivation can well predict good goal performance (Osbaldiston and Sheldon, 2003; Vansteenkiste et al., 2004). We thus argue that, compared with external investigation, self-reporting may lead to a much lower level of budgetary slack.

There is also a chronological order between external investigation and self-reporting, namely, the latter only occurs after the former. Therefore, in this study, we address this issue by formulating into the three different budgeting contexts, including the first budgeting context where no VI is required, the second budgeting context where just large budgetary variance will be investigated by superior (i.e., external investigation which is in line with the inquiry phase of accountability), and the third budgeting context where large budgetary variance will be investigated and the causes of the large budgetary variance are required to be reported by subordinates (i.e., external investigation and self-reporting which are in line with the inquiry and accounting phases of accountability). By exploring the level of budgetary slack in these three budgeting contexts, we attempt to distinguish between accountability pressure generated from external investigation and that from self-reporting, as well as their varying effects on budgetary slack.

Furthermore, prior studies indicate that behavior of budgetary slack is not consistent with the role-related and professional norm (Maiga and Jacobs, 2007a); rather, propensity to create budgetary slack is regarded as a moral problem to organizations’ decision-making (e.g., Douglas and Wier, 2000, 2005; Davila and Wouters, 2005). Such propensity is often linked to different levels of moral development, including ethical concerns (Stevens, 2002), moral equity (Maiga, 2005), moral judgment (Maiga and Jacobs, 2007a), and ethical position (Douglas et al., 2007). Although individual moral development is an important ethical element affecting decision-making in organizational management (Rutledge and Karim, 1999; Schatzberg et al., 2005; Schminke et al., 2005; Ambrose et al., 2007; Maroney and McDevitt, 2008), there is relatively limited understanding as to whether any effects of external investigation and self-reporting on budgetary slack would persist in the subordinates’ decision-making process when subordinates have different levels of moral development.

According to cognitive moral development (CMD) theory, individuals with low level of moral development will make their decisions based on obedience and punishment that stemmed from external rules, while individuals with high level of moral development will make their decisions conditioned by their self-chosen moral principles (Kohlberg, 1969). As aforementioned, external investigation can generate accountability pressure through stipulation of external rules, while self-reporting can generate accountability pressure from both intrinsic principles and external rules. In other words, external investigation may generate more accountability pressure on subordinates with low level of moral development than those with high level of moral development in the context of subordinates’ propensity to create budgetary slack. On the other hand, there may not be any observable moderating effect of moral development on self-reporting and budgetary slack. This is due to the innateness of self-reporting, namely, external rules and self-principles may affect accountability pressure differently. Those subordinates with low level of moral development may respond more to external rules innate in self-reporting, while those with high level of moral development may respond more to self-principles innate in self-reporting. Therefore, we predict that the aggregate moderating effect of moral development on self-reporting and budgetary slack may not be obvious. Thus, in this paper, we attempt to examine the moderating effect of subordinate’s moral development on the respective relationship of external investigation and self-reporting with budgetary slack. We address this issue by using the Defining Issues Test (DIT) to measure the level of subjects’ moral development in our three different experimental budgeting contexts.

In summary, built on prior research on VI and its effect on budgetary slack, this paper aims to explore two issues: (1) the relationships between accountability-oriented control aspects of external investigation and self-reporting and budgetary slack and (2) the moderating effect of moral development on these relationships.

This study makes several significant contributions to the budgetary slack and budget control literature. First, our study contributes to the relatively limited literature on VI at the *ex ante* budgeting process. Second, we provide some new insights into the nature of VI, a common control activity, from the perspective of accountability process. We further reveal that the two inherent control aspects (external investigation and self-reporting) can have varying effects on propensity to create budgetary slack. Finally, we reveal that the level of moral development may have some moderating effects on creating budgetary slack in the context of external investigation and self-reporting. Findings of our study may provide some management ideas on design and implementation of effective budgetary control systems in practice.

This paper proceeds as follows. We develop our hypotheses based on the discussion of the relevant literature in the next section. The third section gives a description of the research method that is employed to test the hypotheses in an experimental setting. The results of the experiment are presented in the fourth section, and the final section discusses the conclusions, the limitations, and some implications for future management control research and practice.

## Hypotheses Development

**FIGURE 1:**
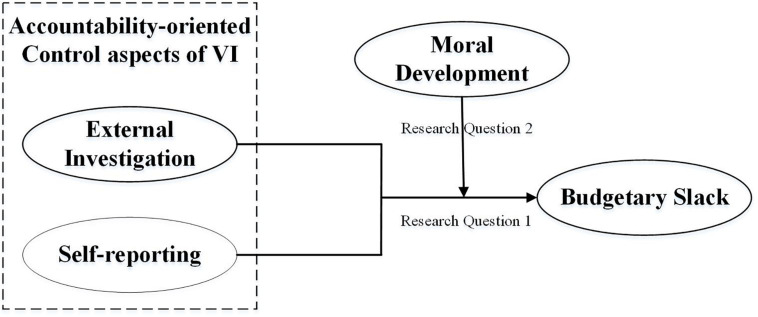
A schematic diagram of this study.

The two research questions discussed above are shown in Figure 1: first, the relationships between accountability-oriented control aspects of external investigation and self-reporting and budgetary slack; second, the moderating effect of moral development on these relationships.

### Accountability-Oriented Control Aspects of VI and Budgetary Slack

Accountability is a process in which individuals should perceive and understand either internal standards (e.g., one’s identity) or external standards (e.g., rules, regulations or law) of behavior, react with these standards, and be answerable to the audiences or oneself (Schlenker and Weigold, 1989). Within management control system research, accountability-oriented control systems are known to influence various levels of organizational hierarchies and antecedents of perceived accountability (Merchant and Otley, 2006). For example, human resource management systems (specially, the “Perceptions of Pay and Promotion”) have a negative effect on the perceived accountability (Hall et al., 2003). The managerial climate of providing feedback to employees directly influences accountability, which directly influences self-development initiatives (Rutkowski and Steelman, 2005). Managerial monitoring of task performance leads to increased perceived accountability for both task performance and interpersonal facilitation, which further results in a positive subsequent performance (Mero et al., 2014).

Similarly, VI, as a type of accountability-oriented control mechanisms, can impose accountability pressure to subordinates, who then feel responsible and obligated to perform certain behaviors that are consistent with external and internal standards. In Webb (2002)’s VI context, control occurs at the stage of rule setting (when the rules are revealed to subordinates) and that of actual actions (at both external investigation and subsequent self-reporting) when there is a “significant” amount of budget variance between the subordinate’s actual capacity known by superior and the budget target submitted by subordinate.

External investigation motivates subordinates to develop a sense of accountability consequential to their actions and therefore regulates their behavior, while self-reporting stimulates subordinates by introjecting such sense of accountability through self-reporting in order to be seen to be in line with external regulations. Those aspects are in line with those demonstrated in respective inquiry and accounting phases of accountability-oriented control (Schlenker and Weigold, 1989). There is also a chorological order, in that inquiry phase only involves external investigation, whereas accounting phase involves both external investigation and self-reporting.

Investigating the large budget variance can impose subordinates with external and specific accountability to create less budget variance at the *ex ante* budget setting stage and in turn induces a clear behavior standard (London et al., 1997). Individuals have the motivation to make a positive impression on the evaluative audience by meeting the perceived behavior standards (Carver, 1979; Webb, 2002). Furthermore, when the specific behavior and the behavior standards of those to whom one is accountable are well defined, the party with accountability is to align their attitudes and behaviors with the known position (Tetlock, 1983; Tetlock et al., 1989). As a result, the accountability pressure generated by VI infuses subordinates to control their motive to create budgetary slack (Webb, 2002).

Both external investigation and self-reporting can generate accountability pressure to reduce subordinates’ propensity to create budgetary slack. Accordingly, we propose the following hypotheses:

H1a. When significant budget variances are to be investigated by superior, namely, external investigation will occur, the level of budgetary slack of subordinates will decrease.

H1b. When the causes of significant budget variances are to be reported by subordinates, namely, self-reporting will occur, the level of budgetary slack of subordinates will decrease.

Although external investigation and self-reporting can both generate perceived accountability pressure, the different characteristics between the two aspects may lead to differences in the subordinates’ perceived accountability pressure.

Under VI policy, subordinates’ perceived accountability pressure from external investigation emerges when they are informed that the superior will conduct an investigation on the “significant” amount of budget variances according to the external standards such as management control policies and rules. Because external standards reflect preferences and performance expectations, subordinates’ perceived accountability pressure will increase when the external standards are high. In addition, audience power is also a key constituent of accountability (Turusbekova, 2007). When the audience is the external superior who monitors, invests, and evaluates the subordinates, the assumed power of the external superior increases subordinates’ perceived accountability pressure. Therefore, the level of subordinates’ perceived accountability pressure generated by external investigation primarily stems from the level of external standards and the implementation of external standards by the external superior, neither of which is controllable by subordinates themselves.

Subordinates’ perceived accountability pressure from self-reporting results from those subordinates who know they will be required to provide an explicit explanation for their “significant” amount of budget variances to the external superior. Just like external investigation, external standards of self-reporting policy and external superior’s power are the main sources of subordinates’ perceived accountability pressure. However, the information quantity and quality (such as authenticity and timeliness) about the cause of “significant” budget variances that subordinates report are mainly influenced by the subordinate’s self-identification and internal principles, which is the related with the process, means, or result of showing oneself to be a particular type of person (Schlenker and Weigold, 1989). Therefore, subordinates’ perceived accountability pressure generated by self-reporting mainly stems from subordinates’ internal self-principles, besides the external standards and external superior’s implementation of the external standards.

Existing literature reveals that perceived accountability pressure has an effect on an individual’s judgment and decision-making (Johnson and Kaplan, 1991; Frink and Ferris, 1998; Tan and Kao, 1999; Hall et al., 2003; Rutkowski and Steelman, 2005; Mero et al., 2014). Some researchers point out that accountability is not a unitary phenomenon (Lerner and Tetlock, 1999; DeZoort et al., 2006). There are different sources of perceived accountability pressure, one from external investigation and the other from self-reporting. According to the self-determination theory, external investigation can be identified as enactment of external regulation, by which a behavior is initiated and maintained by contingencies external to the person. On the other hand, self-reporting can be identified as introjected regulation that motivates people to behave in line with the regulation within oneself and their fragile egos (e.g., Ryan, 1982; Gagné and Deci, 2005). Therefore, although external investigation and self-reporting are related with the controlled motivation, self-reporting can stimulate more internalized motivation than external investigation. Internalized motivation can well predict good goal performance (such as budgetary slack creation) (Osbaldiston and Sheldon, 2003; Vansteenkiste et al., 2004). Therefore, we propose the following hypothesis:

H1c. Self-reporting can cause a much lower level of budgetary slack than external investigation can.

### The Moderating Effect of Moral Development on the Relationship Between Accountability-Oriented Control Aspects of VI and Budgetary Slack

Behavior of budgetary slack is a moral problem within the organizational decision-making process because it can lead to resource misallocation and inconsistency with the role-related and professional norm (Douglas and Wier, 2000, 2005; Maiga and Jacobs, 2007b). Although prior studies find that individual moral development is an important ethical element effecting the decision makers (Rutledge and Karim, 1999; Schatzberg et al., 2005; Schminke et al., 2005; Ambrose et al., 2007; Maroney and McDevitt, 2008), it is unknown whether the effect of accountability-oriented control aspects of VI (external investigation and self-report) on budgetary slack would persist in subordinates’ decision-making process when subordinates have different levels of moral development.

The CMD theory provides a taxonomy of cognitive development including three levels: (1) pre-conventional, (2) conventional, and (3) post-conventional (Kohlberg, 1969). According to CMD, an individual’s moral reasoning in the pre-conventional level is influenced by the punishment and self-interest. In the conventional level, an individual’s moral reasoning focuses on how to maintain good relationship with others, authorities, and social-orders. In the postconventional level, an individual’s moral reasoning turns into individual rights, self-chosen principles, and belief (ibid.). Many studies support that individuals with a low level of moral development will make their decisions based on the obedience and punishment that stemmed from external rules, while individuals with a high level of moral development will make their decisions conditioned by their self-chosen moral principles (Graham, 1995; Patterson, 2001). For example, Ponemon and Gabhart (1990) find that auditors at lower levels of moral reasoning are more sensitive to penalty factors for misconduct than auditors at higher levels of moral reasoning. Kaplan et al. (1997) find that a legal sanction’s communication is most effective in reducing tax evasion intentions for taxpayers at low moral reasoning level.

As aforementioned, external investigation can generate accountability pressure from external rules, and self-reporting can generate accountability pressure from both the self-principles and external rules. Therefore, in line with the taxonomy of moral development levels, external investigation will generate more accountability pressure on the subordinates with a low level of moral development than on those with a high level of moral development. Consequently, external investigation is more effective in creating budgetary slack among subordinates with a low level of moral development than those with a high level of moral development.

However, there is perhaps no moderating effect of moral development on the relationship between self-reporting and budgetary slack. Under the self-report policy, subordinates’ perceived accountability pressure stems from two sources: external rules and self-principles. Subordinates with low moral development respond more to the former, while subordinates with high moral development respond more to the latter. Therefore, when external rules and self-principles are featured together within the self-reporting of VI policy, subordinates with different levels of moral development may have obscure differences in their perceived accountability pressure. As such, there may be no difference in propensity to reduce or create budgetary slack between subordinates with low and high levels of moral development at the self-reporting phase. Accordingly, we propose the following hypotheses:

H2a. Subordinate’s moral development has a moderating effect on the relationship between external investigation and budgetary slack, such that the relationship between external investigation and budgetary slack will be stronger for subordinates with a low level of moral development than for those with a high level of moral development.

H2b. Subordinate’s moral development perhaps has no moderating effect on the relationship between self-reporting and budgetary slack, such that the relationship between self-reporting and budgetary slack will be indifference for subordinates with low or high levels of moral development.

## Experimental Method

### Experimental Overview

In this experiment, we design a working context with different VI aspects in a hypothetical company (ABC Company) (as described in section “Independent Variables”), in which participants are asked to assume the role of an employee to complete a simple letter-decoding task (as described in section “Experimental Procedures”). The task is for participants to translate words from coded letter sequences. Initially developed by Chow (1983), similar forms of the task are commonly used in experimental budgeting research (e.g., Chow et al., 1994; Fisher et al., 2000; Libby, 2001; Webb, 2002). We incorporate an incentive scheme (as described in section “Experimental Procedures”—stage 4) in which participants are paid based on the number of words translated and budget targets that are set by participants themselves. The related operation software and online questionnaire system are designed to simulate the real production and budgeting process. The entire task takes around 60 min to complete on average.

### Measurement of Variables

#### Independent Variables

Independent variables in this study include the two accountability-oriented control aspects of VI (i.e., external investigation and self-reporting). To test the aforementioned hypotheses, we employ a between-subjects design with different accountability-oriented control aspects of VI manipulated randomly in three different budgeting contexts, including no VI context, only external investigation of VI context, and both external investigation and self-reporting of VI context. This experimental design is to simulate the chronological order of the VI process (as explained in section “Introduction”), different to a 2 × 2 full-factorial design commonly employed in experimental research.

Participants in our experiment are randomly divided into three budgeting contexts. Participants are assumed as the employee of ABC Company, and experimental managers are assumed as the manager of ABC Company. In the no VI context, participants are told to submit their budget targets. In other words, the participants have full freedom to determine their budget target.

In the only external investigation context, subordinates know that superiors have the right to conduct an investigation when the amount of budget variance is “significant.” If the budget variances are investigated, the superiors may impose the corresponding penalties to subordinates. In both external investigation and self-reporting context, subordinates are informed that when superiors conduct an investigation for the “significant” amount of budget variances, those subordinates with a large amount of budget variance have to report their reasons for the variances. In short, the difference in budgetary slack between “no VI context” and “only external investigation” is due to the effect of external investigation. The difference in budgetary slack between “only external investigation” and “both external investigation and self-reporting” is due to the effect of self-reporting.

#### Dependent Variable

The dependent variable in this experiment is budgetary slack, which is the difference between the actual production capacity of the subordinate and the amount of the budget target submitted. Similar to the experimental methods used in prior studies (Stevens, 2002; Webb, 2002; Hobson et al., 2011), in order to eliminate the learning effect, participants’ actual production capability is based on their last production record of three training rounds. Similarly, participants’ submitted budget is based on budget target submitted in the last run from the three formal production runs. Therefore, budgetary slack can be expressed by the following formula:

Budgetary Slack = Actual Production Capability - Submitted Budget

#### Moderating Variable

In this study, we use the DIT to measure the moderating variable, moral development of subordinates. DIT was developed by Rest (1979) based on the Kohlberg’s (1969) CMD. DIT as a reliable and valid measure of moral development is supported by many researches (Rest, 1979; Rest and Narváez, 1994; Rest et al., 1997; Thoma and Dong, 2014). The DIT uses several hypothetical moral dilemmas, and each dilemma has 12 statements of issues reflecting the different stages of moral development. Participants are required to consider these moral dilemmas to make an action choice and rank the relative moral importance of each of several standard items for resolving each dilemma. On the basis of item rankings, a DIT *P* score can be derived to reflect the individual’s level of principle moral development, in that individuals with higher *P* scores tend to rely more on the moral principle, which is the characteristic of the postconventional level of moral development. Individuals with lower *P* scores tend to depend on the rule and self-interest, which is the characteristic of the conventional and preconventional level of moral development. Therefore, the level of moral development of individuals with higher *P* scores is relatively higher than individuals with lower *P* scores.

In order to detect participants who do not understand the test or rank the relative moral importance of the items following the moral dilemmas based on their pretentiousness or lofty sound, five essentially meaningless items (M-items) using complex style or verbiage are mixed into each moral dilemma. When a participant ranks too many of these M-items too often, the score of M-items that is calculated as the measuring method of *P* score will be high. If a participant has a score of 8 or more on M-items in a standard version DIT, the data of this participant are assumed as invalid (Rest et al., 1999). In this study, due to time constraints, we use the short version DIT including three moral dilemmas instead of the standard version with six moral dilemmas (e.g., Schatzberg et al., 2005; Kerler and Killough, 2009). Accordingly, we adjust the score of M-items to 4.

### Experimental Procedures

The experimental procedures are shown as follows. At Stage 1, all participants are randomly divided into three groups (i.e., the group with no VI context, the group with only external investigation of VI context, and the group with both external investigation and self-report of VI context) and assigned to three different behavioral research laboratories. In order to avoid any communication among other participants, every participant is put into a cubicle with a computer. The participants are first presented the case information in detail, asking them to assume themselves as subordinates in ABC Company who are about to undertake a production task. The production task is to translate the ASCII code, which translates two-digit numbers into the corresponding letters according to the ASCII rule (for example, “65” = “A,” “66” = “B,”,“90” = “Z”). When participants translate the two ASCII codes successfully each time, one product is produced. We develop computer software to facilitate the experiment. To test their understanding of the production task, participants are required to complete five letter-decoding examples in the control questionnaire 1. Results indicate that all participants can understand the production task.

Stage 2 consisted of three training rounds to provide participants with an opportunity to become familiar with the production task before formal production. The time of each training round is 3 min. At the end of each round, the software will automatically inform the participants of the number of correctly and incorrectly decoded numbers in this round. In line with the method of Stevens (2002) and Hobson et al. (2011), in order to eliminate the learning effect, the actual production capacity of the participant is measured as the number of correctly decodes in the last training round. After the training process, all participants can understand their own actual production capacity privately.

In stage 3, all participants are informed the compensation contract according to the following formula that will be used for the Stage 5 formal production period:

P = 2 + 0.5 (A - B) when A ≥ B

= 2 when A ≤ B

Where P is the total reward obtained by the participant, A is the amount of production in each formal production round, and B is the budget target submitted by participant at the beginning of each formal production round. In other words, the participants are given the basic wage of RMB2. Furthermore, participants make each additional product more than his budget target; he will obtain an additional RMB0.5 for reward. Therefore, this compensation contract has been proved as a slack-inducing pay scheme (Chow et al., 1991), because this can generate a financial incentive for creation of budgetary slack.

In addition, the case materials also present information that participants are required to submit their budget prior to each formal production. Therefore, just as the design of Webb (2002), this experimental design provides subordinates with three conditions necessary for budgetary slack (Dunk, 1993; Dunk and Nouri, 1998): (1) private information (Stage 2); (2) incentive to create budgetary slack (Stage 3); and (3) participated in their budget-setting (Stage 3).

To test their understanding of the compensation contract, subjects are required to answer a question on a seven-point Likert scale (strongly disagree to strongly agree) such as the following item: “Under this compensation contract of ABC Company, given a subordinate’s production capacity, the lower the budget target submitted by the subordinate, the higher the salary is.” Results show that the mean of this item is significantly greater than the scale midpoint (4) [mean = 5.845, standard deviation (SD) = 1.009, *p* < 0.001, two-tailed], which means that subjects understand well the compensation contract.

In stage 4, participants of three groups are provided separately with some different policy about VI in budget setting. In the group with no VI context, participants are not provided with any information about VI.

In the group with only external investigation of VI, participants are told that the experiment manager will conduct an investigation to the subordinates’ budgeting process when there is a “significant” amount of budget variances between subordinate’s actual capacity known by superior and budget targets submitted by this subordinate. Chow et al. (1991) indicate that participants would tend to set a 30% budgetary slack even when the participants’ behavior did not have any consequences. Therefore, 30% is defined as the critical point of this VI policy. In particular, when a participant’s budget variance is equal to or larger than 30% of the average level of budget variance of all participants, the manager will investigate this participant. When the participants are investigated, if they are found that their actual production capacity does not match his budget, the participants would be deducted from the performance salary as punishments.

In the group with both external investigation and self-report of VI context, beside the policy in the group with only external investigation of VI context, participants are provided that they have to report the reasons why the “significant” budget variance occurs. The participant is informed that the superior will consider his report to determine the participant’s final reward or punishment.

To test the effectiveness of our manipulations in this experiment, all participants are required to complete the control questionnaire 3 (described in detail in section “Manipulation Checks”).

Stage 5 is the formal production period with three production rounds. At beginning of each production round, participants are required to submit their own budget target into the decoding software with the consideration of their actual production capacity and the VI policy they are involved. Then, the participants complete the 3-min production task for three rounds.

In stage 6, participants are required to complete the DIT. Then, participants are paid before leaving the laboratory.

### Participants

Debates over the use of students as the surrogate subjects reach some consensus in behavioral accounting research (Walters-York and Curotola, 2000). Further evidence is found that the use of advanced level accounting students is appropriate in the experiment with relatively structured decision settings (Mortensen et al., 2012). This is what is adopted in this experiment; 213 students in Year 3 of a 4-year accounting degree program at a Chinese university participate in this study. Further, the rationale for selecting this group of students is as follows. First, the task employed in this study is simple enough that every participant can have the appropriate matching competency to complete it after necessary training. Second, they are similar in age and cultural and educational backgrounds so that the other factors except variables manipulated in this study may pose less influence to their behavior. Third, they have systematically studied related courses about budget and related budget management topics before the start of the experiment despite lacking practical experience of budgeting in real enterprise. Finally, it is relatively easy to organize such a large group to enable this experiment to be conducted at the same time. Since there are 23 participants whose score of M-items in DIT is 4 or more and 15 participants whose data of DIT are missing, the final sample is 175.

## Results

### Manipulation Checks and Descriptive Statistics

#### Manipulation Checks

Several items are included in control questionnaire 3 to check the effectiveness of our manipulations. All participants are required to answer all manipulation check questions to indicate their agreement with the presented statements on a seven-point Likert scale (strongly disagree to strongly agree). In order to examine the external investigation manipulation, we use the following item: “ABC Company has a policy that superior will conduct an investigation to the subordinate’s budgeting process when there is a ‘significant’ amount of budget variances between the subordinate’s actual capacity known by superior and budget targets submitted by the subordinate.” The mean score on this item was significantly lower in the no VI context than in the only external investigation of VI context (mean = 3.54, SD = 0.828 versus mean = 5.72, SD = 1.14, *p* < 0.001, two-tailed). Meanwhile, there is no significant difference on the mean score on this item in the external investigation compared to both external investigation and self-reporting (mean = 5.72, SD = 1.14 versus mean = 5.50, SD = 1.513, *p* = 0.382, two-tailed). That means our manipulation of external investigation is successful.

To examine the self-reporting manipulation, we use the following item: “ABC Company has a policy that when the investigation is conducted by superior, subordinate with the ‘significant’ budget variance would be required to report the cause of this variance by oneself.” We find that there is no significant difference on the mean score on this item in the no VI context compared to only external investigation of VI context (mean 2.80, SD = 0.654 versus mean = 2.94, SD = 0.712, *p* = 0.272, two-tailed). Meanwhile, the mean score on this item was significantly lower in the external investigation than in both external investigation and self-reporting context (mean = 2.94, SD = 0.712 versus mean = 6.55, SD = 0.769, *p* < 0.001, two-tailed). Therefore, we can confirm that our manipulation of self-reporting is effective.

#### Descriptive Statistics

Table 1 shows descriptive statistics on the budgetary slack and moral development in each of the three different budget contexts. As for budgetary slack, the mean in the no VI context is 13.11 (SD = 9.923). The average budgetary slack of subordinates in only external investigation of VI context is 6.7 (SD = 5.204), and the mean of budgetary slack created by subordinates in both external investigation and self-report of VI is 4.77 (SD = 5.423). These results indicate that the budgetary slack has a downward tendency when the accountability-oriented control characteristics occur in our hypothetical budget contexts.

**TABLE 1:** Descriptive statistics.

	No VI (NV)			Only external investigation of VI (EI)			Both external investigation and self-reporting of VI (ES)			Overall		
	*N*	Mean	SD	*N*	Mean	SD	*N*	Mean	SD	*N*	Mean	SD
Budgetary slack	61	13.11	9.923	54	6.70	5.204	60	4.77	5.423	175	8.27	8.090
Moral development	61	0.308	0.130	54	0.294	0.150	60	0.337	0.151	175	0.314	0.144

In terms of the moral development measured by the *P* score of DIT, the mean of all subordinates’ moral development is 0.314 (SD = 0.130). The mean value of subordinates’ moral development in no VI context is 0.308 (SD = 0.150), that in only external investigation of VI context is 0.294, and that in both external investigation and self-report of VI is 0.337 (SD = 0.151). There is no obvious trend and significant difference among the mean value in the three different budget contexts (ANOVA test, *F*_2_,_172_ = 1.328, *p* = 0.268).

**FIGURE 2:**
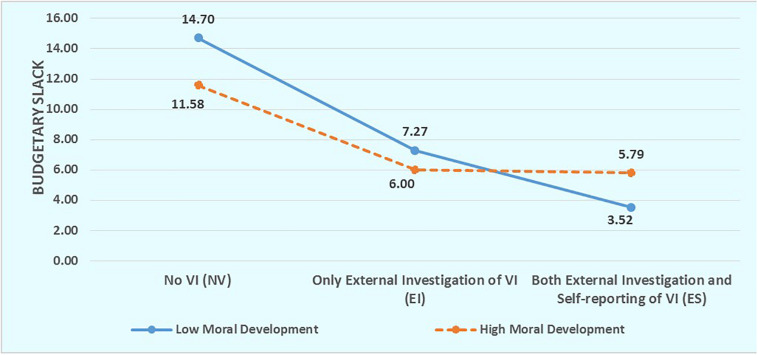
Budgetary slack of subordinates with low and high moral development in the three different budget contexts. The participants with *P* scores less than 0.314 (the mean of *P* score of all participants) are placed in the “Low Moral Development” Group and the other participants are placed in the “High Moral Development” group.

In addition, we divide all participants into the “Low Moral Development” group and the “High Moral Development” group according to the mean of *P* score of DIT (0.314). As shown in Figure 2, there is more obvious decreasing tendency of budgetary slack in the “Low Moral Development” group than in the “High Moral Development” group.

### Hypothesis Test

#### Hypothesis Test About the Relationship Between Accountability-Oriented Control Aspects of VI and Budgetary Slack

Our hypotheses H1a and H1b predict that the accountability-oriented control aspects of VI, external investigation and self-reporting, can reduce the level of budgetary slack of subordinates. Table 2A shows a significant effect of VI on the budgetary slack (*F*_2_,_172_ = 21.775, *p* < 0.001, two-tailed). The results in Table 2B indicate some support for hypotheses H1a and H1b. The mean of budgetary slack created by subordinates in no VI context is significantly higher than that in only external investigation (mean difference = 6.411, *p* < 0.001, two-tailed), which indicates that external investigation has a significant reductive effect on budgetary slack as hypothesis H1a predicts. In addition, the mean of budgetary slack created by subordinates in only external investigation of VI context is significantly higher than that in both external investigation and self-reporting at the 10% level (mean difference = 1.937, *p* = 0.055, two-tailed), which means that the effect of self-reporting on budgetary slack is significant as the prediction of hypothesis H1b.

**TABLE 2:** The effect of accountability-oriented control characteristics of VI on budgetary slack.

(A) One-way ANOVA (means with standard deviations in parentheses)				
	No VI (NV) *n* = 61	Only external investigation of VI (EI) *n* = 54	Both external investigation and self-reporting of VI (ES) *n* = 60	One-way ANOVA
Budgetary slack	13.11 (9.923)	6.70 (5.204)	4.77 (5.423)	***F* = 21.775 *p* < 0.001**
Moral development	0.308 (0.130)	0.294 (0.150)	0.337 (0.151)	*F* = 1.328, *p* = 0.268
**(B)** Mean comparison between three VI contexts (mean difference between group with *p* value in parentheses)				
	**NV vs. EI (effect of external investigation of VI)**		**EI vs.ES (effect of self-reporting of VI)**	
Mean difference	6.411*** (*p* < 0.001)		1.937* (*p* = 0.055)	
**(C)** Different effect of control characteristics of VI on budgetary slack (Dependent variable *Y* is budgetary slack)				
	**Model 1 (Independent variable *X* is a dummy variable; when it is NV, *X* = 0; when it is EI, *X* = 1) (*n* = 115)**		**Model 2 (Independent variable *X* is a dummy variable; when it is EI, *X* = 0; when it is ES, *X* = 1) (*n* = 114)**	
Constant	13.115		6.704	
Coefficients (Std. Error)	-6.411*** (1.506)		-1.937* (0.999)	
*T* value (*p* value)	-4.256 (*p* < 0.001)		-1.939 (*p* = 0.055)	
*F* value (*p* value)	18.116*** (*p* < 0.001)		3.758* (*p* = 0.055)	
Test of difference of these two models’ coefficients	***Z* = -2.476 (*p* = 0.013)**			

***, **, and * indicate 1, 5, and 10% level of significance (two-tailed), respectively. Bold is used to highlight these statistics values.

Our hypothesis H1c predicts that the effect of self-reporting on budgetary slack is stronger than the effect of external investigation. In order to examine this hypothesis, we use the method suggested by Clogg et al. (1995) to compare the regression coefficients between two models in which budgetary slack is the dependent variable. External investigation as a dummy variable is the independent variable in model 1, and self-reporting as a dummy variable is the independent variable in the model 2. The results in Table 2C show that the regression coefficients of VI’s control aspects (i.e., external investigation and self-reporting) are significantly different between two models (*Z* = -2.476, *p* = 0.013, two-tailed). Interestingly, in contrast to the prediction of hypothesis H1c, external investigation has much stronger influence on the budgetary slack than self-reporting has. We will discuss this finding in Section “Conclusions and Implications.”

#### Hypothesis Test About the Moderating Effect of Moral Development on the Relationship Between External Investigation and Self-Reporting and Budgetary Slack

To test our hypothesis about the moderating effect of moral development on this relationship, we use a regression-based path analysis with the aid of existing computational tools to estimate and study the interactions and conditional effect (Hayes and Matthes, 2009; Hayes and Rockwood, 2017). Our hypothesis H2a predicts that the relationship between external investigation of VI and budgetary slack will be stronger among those subordinates with low moral development and weaker among those subordinates with high moral development. The result of Model 1 in Table 3 supports our hypothesis H2a. The regression coefficients of interaction of external investigation and moral development (EI^∗^MR) are significant at the 10% level (regression coefficients = 20.2890, *t* = 1.8930, *p* = 0.0610, two-tailed). In addition, external investigation is still a significant reduction factor of the creation of budgetary slack (regression coefficients = -6.533, *t* = -4.4074, *p* < 0.001, two-tailed) in Model 1, which still support hypothesis H1a.

**TABLE 3:** Ordinary least squares regression to test the moderating effect of moral development on the relationship between accountability-oriented control characteristics of VI and budgetary slack.

	Model 1 (Dependent variable is the budgetary slack) *n* = 115		Model 2 (Dependent variable is the budgetary slack) *n* = 114	
Predictor	Coefficient (*T* value)	*P*	Coefficient (*T* value)	*p*
Intercept	10.1770 (13.7569)	<0.001	5.6449 (11.1547)	<0.001
External investigation of VI (EI)	−6.5330*** (−4.4074)	<0.001		
Self-reporting of VI (SR)			−2.0564** (−2.0287)	0.0449
Moral development (MR)	−9.7218* (−1.8021)	0.0742	2.9792 (0.8872)	0.3769
EI* MR	20.2890* (1.8930)	0.0610		
SR* MR			**3.6841 (0.5475)**	0.5852
Model *R*^2^	0.1823	<0.001	0.0420	0.1915
Interaction Δ *R*^2^	0.0264	0.0610	0.0026	0.5852

***, **, and * indicate 1, 5, and 10% level of significance (two-tailed), respectively. External investigation of VI (EI), self-reporting of VI (SR), and moral development (MR) are mean centered to render parameter estimates that are interpretable within the range of the data. All coefficients are unstandardized and based on models with all primary variables entered. Bold is used to highlight these statistics values.

**FIGURE 3:**
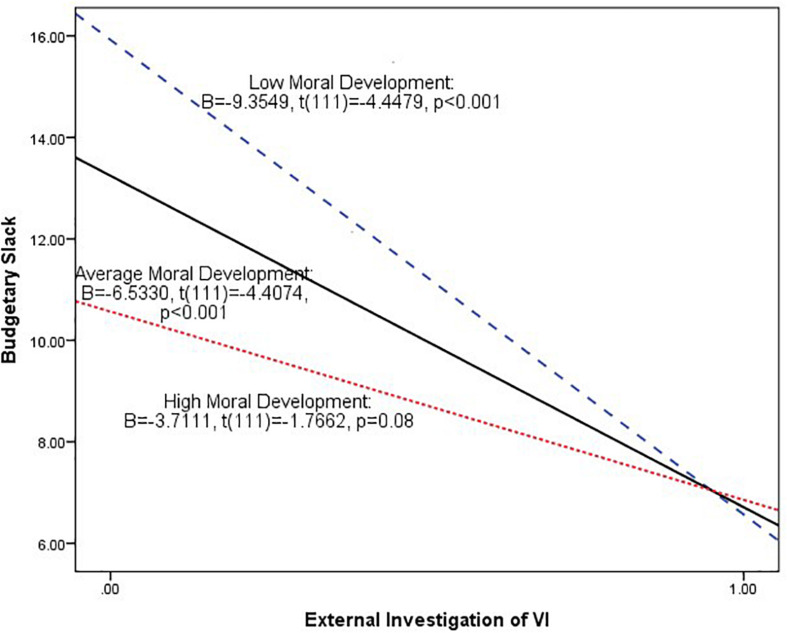
Plot of the predicted value of budgetary slack at external investigation of VI at low, average, and high levels of moral development. The average, low, and high level of moral development are defined according to the mean, mean + 1.0 standard deviation, and mean − 1.0 standard deviation of moral development. The slope of each plotted line is the simple slope of external investigation of VI at the various values of moral development.

After establishing the interaction effects of external investigation and moral development, we then describe this interaction graphically in Figure 3, which plots the conditional effect or “simple slope” of external investigation at various values of moral development (low, average, and high moral development) by using the estimated coefficients from the model. As Figure 3 shows, among the subordinates with low and average moral development, the relationship between external investigation and budgetary slack is significantly negative (low moral development: *B* = -9.9349, *t* = -4.4479, *p* < 0.001 and average moral development: *B* = -6.5330, *t* = -4.4074, *p* < 0.001). Among the subordinates with high moral development, the relationship between external investigation and budgetary slack is also significantly negative at the 10% level (*B* = -3.7111, *t* = -1.7662, *p* = 0.08). As can be seen, the slopes of plotted lines are negative and then appear to flatten as the level of moral development increases.

**FIGURE 4:**
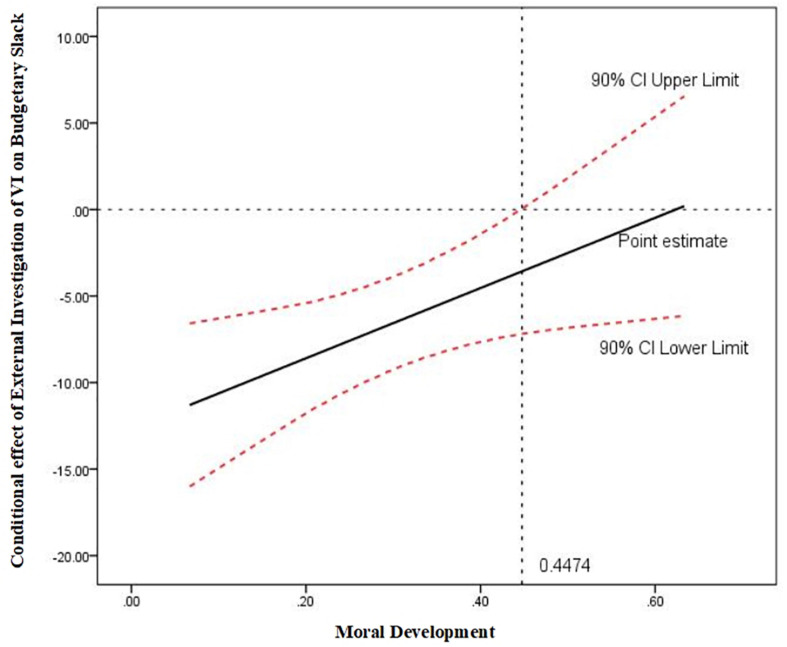
Johnson–Neyman regions of significance for the conditional effect of external investigation of VI on budgetary slack at values of moral development.

Furthermore, we probe this interaction by utilizing the Johnson–Neyman technique (Bauer and Curran, 2005; Hayes and Matthes, 2009). This technique can mathematically derive the “regions of significance” for the conditional effect of moderator, which means the value within the range of the moderator in which the association between dependent variable and independent variable is statistically different from zero (Pollack et al., 2012). Figure 4 shows the conditional effect (the solid line) of external investigation on budgetary slack across the distribution of moral development, as well as the upper and lower bounds of a 90% confidence interval (the dashed lines) for the conditional effect. As shown in Figure 4, when moral development (*P* score of DIT) is less than 0.4474, the effect of external investigation on budgetary slack is significantly negative at the 10% level, whereas when moral development (*P* score of DIT) is greater than 0.4474, there is no evidence of relationship between external investigation and budgetary slack.

After studying the moderating effect of moral development on the external investigation and budgetary slack, we further probe the question whether the relationship between self-reporting and budgetary slack depends on contact with various levels of moral development. As shown in Model 2 in Table 3, the regression coefficients of interaction of self-reporting and moral development (SR^∗^MR) are not significant (regression coefficients = 3.6841, *t* = 0.5475, *p* = 0.5852, two-tailed). This result is consistent with Hypothesis H2b. In addition, the coefficients of self-report are still significantly negative (regression coefficients = -2.0564, *t* = -2.0287, *p* = 0.0449, two-tailed), which still supports hypothesis H1b.

**FIGURE 5:**
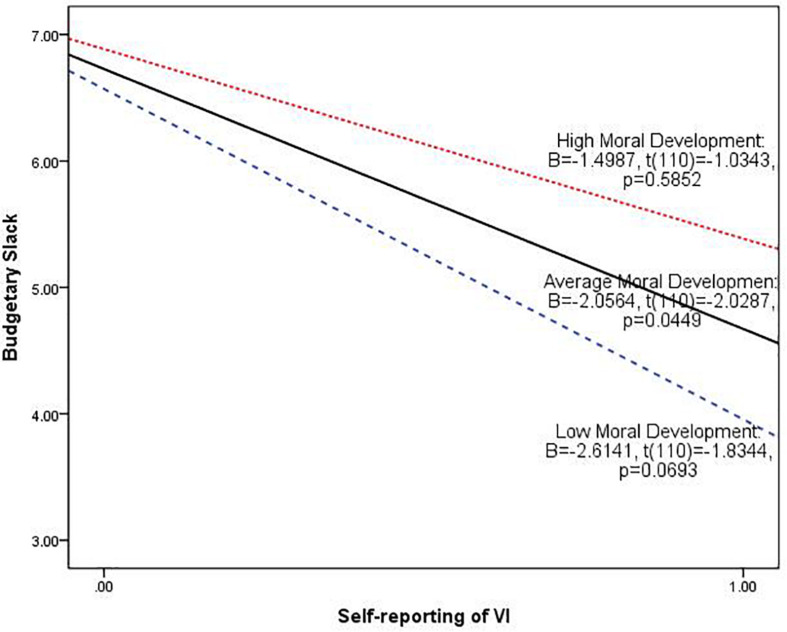
Plot of the predicted value of budgetary slack at self-report of VI at low, average, and high levels of moral development. The average, low, and high level of moral development are defined according to the mean, mean + 1.0 standard deviation, and mean − 1.0 standard deviation of moral development. The slope of each plotted line is the simple slope of self-reporting of VI at the various values of moral development.

The information shown in Figure 5 can help us to probe deeper into the effect of moral development on the relationship between self-reporting and budgetary slack. As can be seen, among the subordinates with low and average moral development, the relationship between self-reporting and budgetary slack is significantly negative at the 10% level (low moral development: *B* = -2.6141, *t* = -1.8344, *p* = 0.0693 and average moral development: *B* = -2.0564, *t* = -2.0287, *p* = 0.0449). However, among the subordinates with high moral development, there is no significant relationship between self-reporting and budgetary slack. These results imply that self-reporting is a complicated control characteristic, and this finding will be further discussed in Section “Conclusions and Implications.”

## Conclusion and Implications

This study has examined in details the relationship between two control aspects of VI (i.e. external investigation and self-reporting) and budgetary slack from the accountability perspective and the moderating effect of moral development on this relationship.

First, our results show that both external investigation and self-reporting can generate the accountability pressure to reduce propensity to create budgetary slack at the *ex ante* budgeting stage (H1a and H1b), which is similar to the argument of Webb (2002). Our study has extended the current understanding of the general effect of VI on budgetary slack to the two control aspects of VI and their varying effects on budgetary slack. As discussed above, we argue that there are different sources of perceived accountability pressure generated by external investigation and self-reporting. In particular, the sources of subordinates’ perceived accountability pressure generated by external investigation is mainly the external standards and their implementation, whereas the sources of subordinates’ perceived accountability pressure generated by self-reporting are both external standards and self-principles.

According to the self-determination theory, self-reporting can stimulate more internalized motivation to control propensity to create budgetary slack than external investigation can. However, our experimental result shows that external investigation has a greater effect on reducing such propensity than self-reporting has, which is contrary to our hypothesis H1c. There are two possible explanations for this result. First, high-internalized motivation (i.e., intrinsic motivation) has more promotional effect on performance in the creative and complicated work setting than in the simple work setting (Amabile, 1985; Ilardi et al., 1993; Koestner and Losier, 2002). Although self-reporting can stimulate more internalized motivation than external investigation, the internalized motivation of self-reporting may not work in our experimental job design which is just a simple letter-decoding task. Second, this result may also be attributed to varying levels of accountability salience exhibited between external investigation and self-reporting. Accountability salience describes the extent to which individuals are held accountable for important outcomes (Hall et al., 2007). When individuals believe that their decisions or actions have more accountability salience, they will perceive more accountability pressure and take more efforts to deal with them. This result may indicate that accountability salience of external investigation is greater than that of self-reporting.

Second, we have found that moral development moderates the effect of external investigation on budgetary slack. In particular, the effect of external investigation on budgetary slack is stronger among the subordinates with low moral development than among those with high moral development. This result is similar to the augmentation of some existing studies (Rutledge and Karim, 1999; Herron and Gilbertson, 2004; Maroney and McDevitt, 2008). For example, Rutledge and Karim (1999) find out that managers are likely to continue an unprofitable project only when adverse selection conditions are present and moral reasoning level is low. Maroney and McDevitt (2008) find out that the perceived influence of the Sarbanes–Oxley Act has a greater effect on the amount of loss recognized in FSA decisions for those participants at lower levels of moral reasoning. Herron and Gilbertson (2004) find the moderating effect of moral development on auditors’ independence judgments.

In addition, in this study, external investigation can be regarded as external imposed control, and moral development (i.e., moral consideration in moral decision-making) can be regarded as self-control. This study finds out that the interaction of external investigation and moral development is substitutable, which is consistent with existing literature. Fishbach and Trope (2005) find out that externally imposed control and self-control are substitutable means for pursuing activities with long-term benefits and short-term costs. Especially in budget research, Webb (2002) also suggests that VI and reputation concerns may serve as cost-effective substitutes to reduce budgetary slack.

Furthermore, this study shows that moral development has no moderating effect on the relationship between self-reporting and budgetary slack. This finding may indicate the complex nature relating to the proportion of perceived exogenous and endogenous accountability pressure generated by self-reporting. When the proportion of perceived exogenous accountability pressure is greater than the endogenous one, subordinates with low moral development will pay no attention to the self-reporting policy, whereas those with high moral development will be sensitive to the self-reporting policy when the proportion of perceived endogenous accountability pressure is greater. That is, there are two opposite effects of moral development on the relationship between self-reporting and budgetary slack.

Unfortunately, since we cannot identify the proportion of external sources (from external investigation) and self-sources (innate principles) of perceived accountability pressure generated by self-reporting, the moderating effect of moral development on the relationship between self-reporting of VI and budgetary slack is not obvious.

Our findings offer some practical implications for management control purposes. First, our study shows that two accountability-oriented control characteristics of VI can both reduce budgetary slack, but external investigation has a much stronger influence on budgetary slack than self-reporting has in our simple work setting. This result suggests that specifically at the *ex ante* budgeting stage, VI is also an effective mechanism of MCS to control propensity to create budgetary slack. The result also suggests that when the task is simple, to reduce budgetary slack, the manager can rely more on external control mechanisms such as external investigation, monitoring, and punishment. Second, our study shows that moral development moderates the relationship between external investigation and budgetary slack. This result suggests that the manager should take into account the subordinates’ level of moral development when they design and implement the specific external control mechanism of MCS (Church et al., 2018).

## Limitations

Of course, our study is subject to several limitations. First, our study argues that VI can generate accountability pressure, which in turn reduces propensity to create budgetary slack. That means accountability pressure has a mediator role between VI and budgetary slack. Consistent with Webb (2002), our study does not empirically test the mediating effect of accountability pressure. In future research, we may examine the differences of perceived accountability pressure between external investigation and self-reporting, and its mediating effect on the relationship between these two control characteristics and budgetary slack. Furthermore, budget participants are concerned with the budget procedure design fairness as well as budget procedure implementation fairness (Magner et al., 2006). Budget participants often have stronger reactions toward the fairness of the budget procedures than the budget outcomes (Little et al., 2002; Maiga and Jacobs, 2007b). Therefore, we can also examine the mediating effect of fairness perception between VI and budgetary slack in future studies (e.g., Church et al., 2018). Another limitation of our study is that we employ two independent variables (external investigation and self-reporting) by using between-subjects design in three different budgeting contexts rather than the 2 × 2 full-factorial design. This research design attempts to simulate the chronological order of the external investigation and self-reporting in VI; it may, however, limit our research on the interactional effect of these two aspects on budgetary slack.

## Data Availability Statement

The raw data supporting the conclusions of this article will be made available by the authors, without undue reservation.

## Ethics Statement

The studies involving human participants were reviewed and approved by the Ethics Committee of Nanjing University of Science and Technology (School of Economics and Management). Written informed consent for participation was not required for this study in accordance with the national legislation and the institutional requirements.

## Author Contributions

DD contributed to design of the study, data analysis, and manuscript writing and revising. LL and SW contributed to manuscript writing and revising. All authors contributed to the article and approved the submitted version.

## Conflict of Interest

The authors declare that the research was conducted in the absence of any commercial or financial relationships that could be construed as a potential conflict of interest.

## Footnotes

1 During the inquiry phase, individuals know the possible occurrence of an evaluation reckoning and judgment, or at least anticipate, in which one’s behavior is compared to certain standards for performance. In the accounting phase, individuals anticipate having to describe, document, interpret, and explain relevant information, thereby reporting a personal version of the events and why they occurred. In the judgment phase, other individuals (or other group, organization) make a verdict based on the information and the comparison of the conduct with the prescription. In the sanction phase, rewards or punishments are then distributed (Schlenker and Weigold, 1989).

2 The external investigation means that an external supervisor would gather information for further decisions by conducting an investigation to subordinates’ budgeting process when there is a “significant” amount of budget variances between subordinate’s actual capacity known by superior and budget targets submitted by subordinate. Thereafter, when external investigation was conducted, subordinate with the large budget variance would be required to indicate in writing the cause of this variance (Webb, 2002, p. 370), hereinafter, termed as “self-reporting.”

3 Webb’s (2002) seminal study only examines the effect of VI as a whole accountability-oriented control mechanism on the budgetary slack.
